# *PIK3CA* mutations in endocrine-resistant breast cancer

**DOI:** 10.1038/s41598-024-62664-1

**Published:** 2024-05-31

**Authors:** Caroline Schagerholm, Stephanie Robertson, Hosein Toosi, Emmanouil G. Sifakis, Johan Hartman

**Affiliations:** 1 Department of Oncology-Pathology, Karolinska Institutet, Bioclinicum, Stockholm, Sweden; 2 Division of Computational Science and Technology, KTH Royal Institute of Technology and Science for Life Laboratory, Stockholm, Sweden; 3 Department of Clinical Pathology and Cancer Diagnostics, Karolinska University Hospital, Stockholm, Sweden

**Keywords:** Breast cancer, Endocrine-resistance, PIK3CA, Breast cancer, Cancer genomics

## Abstract

Around 75% of breast cancer (BC) patients have tumors expressing the predictive biomarker estrogen receptor α (ER) and are offered endocrine therapy. One-third eventually develop endocrine resistance, a majority with retained ER expression. Mutations in the phosphatidylinositol bisphosphate 3-kinase (PI3K) catalytic subunit encoded by *PIK3CA* is a proposed resistance mechanism and a pharmacological target in the clinical setting. Here we explore the frequency of *PIK3CA* mutations in endocrine-resistant BC before and during treatment and correlate to clinical features. Patients with ER-positive (ER +), human epidermal growth factor receptor 2 (HER2)-negative primary BC with an ER + relapse within 5 years of ongoing endocrine therapy were retrospectively assessed. Tissue was collected from primary tumors (*n* = 58), relapse tumors (*n* = 54), and tumor-free lymph nodes (germline controls, *n* = 62). Extracted DNA was analyzed through panel sequencing. Somatic mutations were observed in 50% (31/62) of the patients, of which 29% occurred outside hotspot regions. The presence of *PIK3CA* mutations was significantly associated with nodal involvement and mutations were more frequent in relapse than primary tumors. Our study shows the different *PIK3CA* mutations in endocrine-resistant BC and their fluctuations during therapy. These results may aid investigations of response prediction, facilitating research deciphering the mechanisms of endocrine resistance.

## Introduction

Breast cancer is the most common malignant disease globally with an increased incidence in all age groups over the last decades^1,2^. Around 75% of patients with breast cancer have tumors expressing estrogen receptor α (ER) and are offered adjuvant endocrine therapy for 5–10 years, which reduces the risk of recurrence by almost 50% and the mortality by up to 30%^3^. However, around 30% of these patients later develop endocrine resistance with progression or relapse in their disease, predominantly with sustained ER expression^4^. Differential pathway signaling of ER and several downstream systems have been investigated, and one of the most prominent pathways is that of phosphatidylinositol 3-kinase/protein kinase B/mammalian target of the rapamycin (PI3K/AKT/mTOR)^5^. PI3K is a family of lipid-kinase enzymes involved in ubiquitous molecular pathways such as proliferation, cell signaling, and metabolism^6,7^, and the pathway has been shown, even in the absence of estrogens, to drive reactivation of ER transcription^8^.

Mutational activity in the *PIK3CA* gene, coding for the catalytic subunit p110α of PI3K, causes the constitutive activation of PI3K and has been suggested as a mechanism for endocrine resistance^6^. In fact, *PIK3CA* mutations are some of the most common genetic variants in the cancer genome, present in up to 40% of ER-positive and human epidermal growth factor receptor 2 (HER2)-negative (ER + /HER2-) breast tumors^6,7^. The presence of *PIK3CA* mutations has appeared to be both an individual negative prognostic factor and a negative predictive factor to chemotherapy treatment in patients with metastasized ER + /HER2- breast cancer^9–11^. Although, studies have also shown associations with positive prognostic factors such as increased age, ER positivity, and smaller tumor size^12^. Furthermore, the spread to regional lymph nodes is one of the strongest prognostic factors in breast cancer^1,13^, and an association between the presence of *PIK3CA* mutations and lymph node metastasis has been suggested^14^. However, associations with other tumor characteristics such as tumor grade and histologic subtype are inconclusive^15,16^.

The PI3K inhibitor alpelisib has been approved by the US Food and Drug Administration and is in the clinical setting in Sweden offered to patients with advanced breast cancer harboring a *PIK3CA* mutation^1^. Alpelisib selectively inhibits the p110α isoform 50 times more effectively than other isoforms^6,17^. Data from the SOLAR-1 trial has shown a clear benefit in terms of prolonged progression-free survival in patients with *PIK3CA* mutated advanced breast cancers treated with both alpelisib and fulvestrant, compared to fulvestrant alone^17^. Though, from the majority of patients (77%) in this study, only non-treated primary tumor biopsies were analyzed and not the metastatic lesion^18^. A meta-analysis comparing *PIK3CA* status between primary and metastatic tumor samples found discrepancies between these in around 10% of the paired samples^19^, suggesting that testing of the metastatic tumor sample is necessary.

The most frequent protein residue changes occur on Glu542Lys, Glu545Lys, and His1047Arg, accounting for almost 70% of *PIK3CA* mutations^6,7^. These are in turn located on the coding exons 9 and 20, corresponding to the C-terminal of the helical and kinase domains of p110α^6,7^. Therascreen® (Qiagen, Hilden, Germany) is one of the tests approved for clinical use to assess *PIK3CA* mutations for inhibitor treatment and was used to detect mutations in the SOLAR-1 trial^17,20^. The test includes the 11 well-described hotspot regions on coding exons 7, 9, and 20^20^. Recent studies have implied the prevalence of several other mutation regions that might be of interest for the introduction of PI3K inhibitors, and the American Society of Clinical Oncology (ASCO) guidelines recommend next-generation sequencing (NGS) if the hotspot mutations are not found^21–23^. It has also been indicated that patients possessing tumors with multiple *PIK3CA* mutations may respond more favorably to PI3K inhibitors if the mutations occur on the same allele, but not on the opposite^24^. This suggests a *PIK3CA*-hypermorphic phenotype where a weakly oncogenic mutation is synergized with a moderately oncogenic mutation, creating a more oncogenic and inhibitor-sensitive phenotype^24^. The above-mentioned studies showed that approximately 20% of patients harboring a *PIK3CA* mutation would go unnoticed by the Therascreen® test^21,22^, and 95% with known double mutations would not be captured^21^. It has not been validated if patients harboring mutations outside of hotspot regions may benefit from inhibitor treatment, though, one of the studies demonstrated improved progression-free survival for such patients treated with alpelisib and fulvestrant as compared to fulvestrant alone,^22^.

The utilization of PI3K-targeted therapy and diagnostic tests is promising, but some uncertainties remain. The frequency of *PIK3CA* mutations in relapsed endocrine-resistant breast cancer and how these may fluctuate compared to primary tumors is unknown. Also, the relationship between the mutations and clinical characteristics remains ambiguous. Moreover, discrepancies in diagnostic testing, what mutational regions to include, and on which samples the tests should be performed, call for further investigation. The overall aim of this study was to investigate *PIK3CA* mutations in endocrine-resistant breast cancer tumors to improve our understanding of the endocrine resistance mechanism. More specifically, we aimed to investigate the mutational status, assess the type and frequency of single-nucleotide variations (SNVs) together with their respective variant allele frequencies (VAFs), and copy number variations (CNVs) in paired primary and relapse tumors prior to and during ongoing endocrine therapy. Furthermore, we aimed to compare clinicopathological characteristics in relation to the mutational status.

## Methods

### Study design

This study was designed as a retrospective cohort study of patients with endocrine-resistant breast cancer diagnosed in 2008–2012 in Stockholm, Sweden. Primary and relapse tumors from the same patient were identified from the pathology laboratory information system at Karolinska University Hospital, Sweden. Tumor-free lymph nodes for each patient were utilized as normal germline samples. Clinicopathological data, treatment information, eventual therapy changes, side effects, and outcome data with over 5 years of follow-up were obtained from electronic medical records. The primary outcome variable was overall survival and the secondary outcome was breast cancer-specific survival (BCSS). The survival endpoints time to event were classified as time from diagnosis to either the time to the event or the follow-up date and the event classified as death by any cause or breast cancer-specific death as stated in the medical records.

### Cohort description

The inclusion criteria were patients with breast cancer with an ER + /HER2- primary tumor that experienced an ER + tumor relapse within five years of ongoing endocrine therapy, thus defined as endocrine-resistant. In this study, intrinsic and acquired resistance was not separately assessed by examining other eventual mutations such as *ESR1* (estrogen receptor 1) and *TP53* (tumor protein P53) but is intended for future analysis of the cohort in an ongoing study. Exclusion criteria involved prior breast cancer, bilateral breast cancer, stage IV disease at diagnosis, neoadjuvant therapy, HER2 + primary tumors, and primary tumor size < 5 mm. Keywords for compliance to therapy were searched for in patient journals, such as tolerance and side effects. Patients who did not adhere to their endocrine treatment were excluded from the study (Supplementary Figure S1). Relapse tumors were categorized as ipsilateral locoregional recurrences (in breast or axilla), contralateral recurrences, and distant metastases. Contralateral recurrent tumors were divided into primary resistant tumors (potentially new breast cancer clones) defined as invasive cancer with ductal carcinoma in situ (DCIS) or secondary resistant tumors without DCIS.

### Tumor tissue collection

Archived formalin-fixed paraffin-embedded (FFPE) blocks with corresponding hematoxylin and eosin (HE)-stained tumor glass slides were retrieved from both primary and relapse tumors, along with tumor-free lymph nodes. Invasive tumor regions from each tumor tissue block were identified and macro dissected, and sections were stored in microcentrifuge tubes at 8 °C. From a few patients, blocks were missing and thus excluded.

### Sub-cohorts

The patients were initially collected in two cohorts; a first cohort (cohort I) assessed in the years 2010–2011 and a second cohort (cohort II) assessed in 2009 and 2012. Cohort I was established to test the hypothesis and methodology, and cohort II comprised further inclusion of patients, validating the findings. In cohort I, patients who had received chemotherapy were initially excluded. However, the interference of treatment effects several years prior to the relapse tumor was re-evaluated as minor and the excluded patients from cohort I were later added to cohort II, further consisting of patients assessed in 2009 and 2012 both treated with and without chemotherapy. The two cohorts were merged in the final analysis.

### DNA extraction and panel sequencing

DNA extraction of cohort I was carried out using the Qiagen QIAamp DNA FFPE Tissue Kit. For cohort II, the Qiagen AllPrep DNA/RNA FFPE Kit was used for extraction. Quality control, library preparation, and panel sequencing were carried out by the core facility Clinical Genomics at SciLifeLab, Stockholm, Sweden. The panel consists of 370 genes, has a size of 2.4 Mb, and is intended for genomic screening of solid tumors^25,26^. Samples that failed library preparation were excluded from the analysis.

### Included study samples

The included study material consisted of a total of 62 patients, comprising their primary tumors (*n* = 58) and relapse tumors (*n* = 54); 18 patients from cohort I and 44 from cohort II. 50 of these patients made up a fully matched pair of primary and relapse tumors, eight patients had orphan primary tumors, and four patients had orphan relapse tumors (Supplementary Fig. 1). The relapse locations involved 22 ipsilateral recurrences, five contralateral recurrences without DCIS, 14 contralateral recurrences with DCIS, and 21 distant metastases (Supplementary Table S1).

### Data analysis of sequencing data

The raw data from the DNA sequencing was received as fastq-files and analyzed through a customized pipeline using Python 3.8.12^27^. Sample data was trimmed by Cutadapt (v 4.1)^28^, and aligned to the reference genome using Burrows-Wheeler Aligner (BWA) (v 0.7.17)^29^. Genome Analysis Toolkit (GATK) (v 4.2.6.1)^30^ was used for the unique molecular identifier (UMI) aware duplicate read marking and base quality score recalibration with Mutect2^31^ for somatic mutation calling. GRCh37/Hg19 was utilized as the reference genome, which was also used for the panel construction^25,26^. Filtration was set to a minimum of 50 read depth and VAF cut-off of > 5% in either primary or relapse tumor samples. The tumor samples were compared to the germline lymph node samples to ensure somatic alterations. Comparisons between the relapse and primary tumor of the same patients were assessed together with multiple mutation analysis, hotspot analysis, and exon location annotation. To compare the fraction of cells carrying each SNV in each pair of primary and relapse samples, the VAF of the mutation in each sample was divided by the estimated tumor content of the sample.

Intronic variants, downstream gene variants, and untranslated region (UTR) variants were excluded. For assessment of the clinical implications of the mutations, Combined Annotation-Dependent Depletion (CADD) scores, which use machine learning models to score SNVs, insertions, and deletions^32^ along with annotations from Ensembl Variant Effect Predictor (VEP) (v 108)^33^, were added for each somatic point mutation^34^. The cut-off for CADD scores was set to ≥ 0.20, for assessing pathogenicity. Further, manual curation of non-hotspot mutations was evaluated through the COSMIC (Catalogue Of Somatic Mutations In Cancer) (v92) database^35^, the dbSNP (the Single Nucleotide Polymorphism Database) (v 135)^36^, and ClinVar^37^ following identification numbers and information from the pipeline analysis. Copy number analysis was carried out on all samples using the Fraction and Allele-Specific Copy Number Estimates from Tumor Sequencing (FACETS) (v 0.6.2) tool^38^. FACETS was run with maximum read coverage increased to 20,000 to account for the deep sequencing in this project. Definitions were set to cut-offs of copy numbers defining 0 as total/deep loss, < 2 as loss, 2 in one allele as CNLOH, 2 in both alleles as neutral, 3–5 as gain, and > 6 as amplification, as based on previous papers^38,39^. Visualization for SNVs and CNVs was carried out in Plotly version 5.12^40^.

### Data analysis of clinicopathological factors

Tumor-infiltrating lymphocytes (TILs) were analyzed from the HE-stained tumor slides of both primary and relapse tumors by board-certified pathologists according to international guidelines from Denkert et al.^41^ and Salgado et al.^42^. For comparisons, immunohistochemistry biomarker status for the proliferation-associated marker Ki67 was defined by either a cut-off of > 15% or > 20% for high Ki67. Progesterone receptor (PR) status was defined by cut-offs of either > 10% and > 20%. According to the guidelines at the point of diagnosis, HER2 status was defined as negative for all patients with immunohistochemistry (IHC) score of 0 and 1 + and for score 2 + and 3 + with negative in situ hybridization (ISH), and as positive for IHC score 2 + and 3 + with positive ISH. For this study, HER2 status was divided into HER2-negative/zero for all patients with HER2 IHC score 0, HER2-low for HER2 IHC scores 1 + to 3 + without gene amplification by HER2 ISH, and HER2-positive if gene amplified by HER2 ISH analysis^43^. The Fisher exact test and the Mann–Whitney U test were used to assess any differences in clinicopathological characteristics for categorical and continuous variables in the patients harboring a mutation versus those that did not in each tumor setting, respectively. All statistical tests applied were two-sided, and a *p*-value < 0.05 was considered statistically significant. Overall survival and BCSS was assessed with the Kaplan–Meier method in packages survival and survminer in R computing environment version 4.2.3^44^. Multivariate analyses were not performed. Calculations, assessments, and visualizations of the clinicopathological data were carried out in Microsoft® Excel® for Microsoft 365 MSO (Version 2303, Build 16.0.16227.20202) 32-bit^45^ and in R^44^.

### Ethical considerations

The study was performed in accordance with the Declaration of Helsinki. The study was approved by the Regional Ethical Review Board in Stockholm, Sweden. Patients provided informed consent prior to surgery for storage of tissue samples in the Stockholm Medical Biobank for clinical and research purposes. No additional informed consent was required in accordance with ethical approval in this non-interventional data collection.

## Results

### Clinicopathological characteristics across *PIK3CA* mutational status

We assessed the *PIK3CA* mutational status in relation to clinicopathological characteristics of primary tumors (Table 1 and Table 2) and relapse tumors (Supplementary Table S1). A significant difference in nodal status was observed in patients with *PIK3CA* mutations compared to those without mutations in the primary setting (*p* = 0.000088; Table 1), as further described below. There was a significant difference in the distribution of adjuvant chemotherapy where a higher frequency of the patients with *PIK3CA* mutations in primary tumors had received the treatment versus those without mutations (*p* = 0.0079; Table 2). This difference was not seen in the comparison of adjuvant chemotherapy to relapse mutational status (*p* = 0.061; Supplementary Table S1) nor in the hotspot mutational settings. There was a significant difference in Ki67 score (*p* = 0.018) between primary tumors with *PIK3CA* mutations (median 15%) compared to those without mutations (median 24%; Table 1 and Supplementary Fig. S2a). However, there was no statistically significant difference in Ki67 status (cut-off 15% or 20%) between primary tumors with versus (vs) without mutation. No statistical differences in the distribution of other clinicopathological variables such as age, histologic grade, tumor size, and HER2 status were observed between primary tumors with vs without *PIK3CA* mutation (Table 1). There was neither significance when assessing TIL scores compared to mutational status in the primary or relapse tumors (*p* = 0.90 and *p* = 0.61, respectively) nor to hotspot status.

**Table 1:** Clinicopathological characteristics for all patients’ primary tumors, as well as for the subgroup with *PIK3CA* mutation and the subgroup with no *PIK3CA* mutation.

	All	Primary tumor with *PIK3CA* mutation	Primary tumor with no *PIK3CA* mutation	P-value mutation (mutation vs no mutation per characteristic)
	N = 62*	N = 23**	N = 35**	
Tumor size				*P* = 0.30
Median, mm (range)	24 (10–100)	25 (10–60)	24 (10–100)	
Tumor stage				*P* = 0.84
pT1	23 (37.10%)	7 (30.43%)	14 (40.00%)	
pT2	34 (54.84%)	14 (60.87%)	18 (51.43%)	
pT3	5 (8.06%)	2 (8.70%)	3 (8.57%)	
pN status				*P* = 0.000070
pN0	35 (56.45%)	6 (26.09%)	28 (80.00%)	
pN1	15 (24.19%)	11 (47.83%)	3 (8.57%)	
pN2	8 (12.90%)	4 (17.39%)	4 (11.43%)	
pN3	4 (6.45%)	2 (8.70%)	0 (0.00%)	
Lymph node status				*P* = 0.000088
Negative	35 (56.45%)	6 (26.09%)	28 (80.00%)	
Positive	27 (43.55%)	17 (73.91%)	7 (20.00%)	
TNM stage				*P* = 0.089
Stage 1	16 (25.81%)	3 (13.04%)	13 (37.14%)	
Stage 2	34 (54.84%)	14 (60.87%)	18 (51.43%)	
Stage 3	12 (19.35%)	6 (26.09%)	4 (11.43%)	
Histological subtype				*P* = 0.75
Ductal	43 (69.35%)	15 (65.22%)	26 (74.29%)	
Lobular	14 (22.58%)	6 (26.09%)	6 (17.14%)	
Other	5 (8.06%)	2 (8.70%)	3 (8.57%)	
Histologic grade				*P* = 0.21
NHG1	9 (14.52%)	5 (21.74%)	4 (11.43%)	
NHG2	28 (45.16%)	12 (52.17%)	15 (42.86%)	
NHG3	24 (38.71%)	5 (21.74%)	16 (45.71%)	
NA	1 (1.61%)	1 (4.35%)	0 (0.00%)	
ER, %				*P* = 0.64
Median, % (range)	90 (5–100)	90 (50–100)	90 (5–100)	
PR, %				*P* = 0.35
Median, % (range)	40 (0–100)	60 (0–100)	40 (0–100)	
PR status				*P* = 0.31
Negative (< 10%)	14 (22.58%)	3 (13.04%)	9 (25.71%)	
Positive (≥ 10%)	35 (56.45%)	16 (69.57%)	18 (51.43%)	
NA	13 (20.97%)	4 (17.39%)	8 (22.86%)	
PR status				*P* = 0.34
Negative (< 20%)	16 (25.81%)	4 (17.39%)	10 (28.57%)	
Positive (≥ 20%)	33 (53.23%)	15 (65.22%)	17 (48.57%)	
NA	13 (20.97%)	4 (17.39%)	8 (22.86%)	
Ki67, %				*P* = 0.018
Median, % (range)	20 (1–95)	15 (1–80)	24 (1–95)	
Ki67 status				*P* = 0.24
Low (< 15%)	20 (32.26%)	9 (39.13%)	9 (25.71%)	
High (≥ 15%)	34 (54.84%)	11 (47.83%)	23 (65.71%)	
NA	8 (12.90%)	3 (13.04%)	3 (8.57%)	
Ki67 status				*P* = 0.090
Low (< 20%)	25 (40.32%)	12 (52.17%)	11 (31.43%)	
High (≥ 20%)	29 (46.77%)	8 (34.78%)	21 (60.00%)	
NA	8 (12.90%)	3 (13.04%)	3 (8.57%)	
HER2 IHC				*P* = 0.17
0	37 (59.68%)	16 (69.57%)	18 (51.43%)	
1 +	11 (17.74%)	4 (17.39%)	6 (17.14%)	
2 +	9 (14.52%)	1 (4.35%)	8 (22.86%)	
3 +	2 (3.23%)	0 (0.00%)	2 (5.71%)	
NA	3 (4.84%)	2 (8.70%)	1 (2.86%)	
HER2 status***				*P* = 0.097
HER2-negative/zero	37 (59.68%)	16 (69.57%)	18 (51.43%)	
HER2-low	22 (35.48%)	5 (21.74%)	16 (45.71%)	
HER2-positive	0 (0.00%)	0 (0.00%)	0 (0.00%)	
NA	3 (4.84%)	2 (8.70%)	1 (2.86%)	
TIL score				*P* = 0.90
Median, % (range)	5 (1–40)	5 (1–40)	5 (1–35)	

ER = estrogen receptor, IHC = immunohistochemistry, ISH = in situ hybridization, NHG = Nottingham Histologic Grade, pN = pathological nodal status, PR = progesterone receptor, TIL = tumor infiltrating lymphocyte, TNM = tumor, nodal, metastasis staging, NA = data not available.

* All patients included, even when lacking sequencing data from the primary tumor.

** Representing the primary tumors where sequencing data could be generated and evaluated.

*** HER2-negative/zero = HER2 IHC score 0, HER2-low = HER2 IHC score 1–3 + and negative HER2 ISH, HER2-positive = HER2 amplified by ISH.

**Table 2:** Patient and treatment information for all patients' primary tumors, as well as for the subgroup with *PIK3CA* mutation and the subgroup with no *PIK3CA* mutation.

	All	*P*rimary tumor with *PIK3CA* mutation	Primary tumor with no *PIK3CA* mutation	P-value mutation (mutation vs no mutation per characteristic)
	N = 62*	N = 23**	N = 35**	
Age at diagnosis				*P* = 0.42
Median, years (range)	61 (30–88)	63 (32–80)	60 (30–88)	
Surgical procedure				*P* = 0.79
Mastectomy	30 (48.39%)	12 (52.17%)	16 (45.71%)	
Partial mastectomy	31 (50.00%)	11 (47.83%)	18 (51.43%)	
NA	1 (1.61%)	0 (0.00%)	1 (2.86%)	
Adjuvant endocrine therapy				*P* = 0.055
Tamoxifen	33 (53.23%)	8 (34.78%)	23 (65.71%)	
Aromatase inhibitor	24 (38.71%)	12 (52.17%)	10 (28.57%)	
Other	5 (8.06%)	3 (13.04%)	2 (5.71%)	
Adjuvant chemotherapy				*P* = 0.0079
Received	32 (51.61%)	17 (73.91%)	13 (37.14%)	
Not received	30 (48.39%)	6 (26.09%)	22 (62.86%)	
Adjuvant radiotherapy				*P* = 0.79
Received	40 (64.52%)	15 (65.22%)	21 (60.00%)	
Not received	22 (35.48%)	8 (34.78%)	14 (40.00%)	
Relapse location				*P* = 0.19
Ipsilateral	22 (35.48%)	6 (26.09%)	15 (42.86%)	
Contralateral without DCIS	5 (8.06%)	2 (8.70%)	2 (5.71%)	
Contralateral with DCIS	14 (22.58%)	4 (17.39%)	10 (28.57%)	
Distant metastasis	21 (33.87%)	11 (47.83%)	8 (22.86%)	

DCIS = ductal carcinoma in situ, NA = data not available.

* All patients included, even when lacking sequencing data from the primary tumor.

** Representing the primary tumors where sequencing data could be generated and evaluated.

Significant differences were seen in the primary tumor Ki67 score (*p* = 0.028) between patients with *PIK3CA* mutations in their relapse tumors (median 14.5%) as compared to those without mutations (median 20%; Supplementary Table S1 and Supplementary Fig. S2b). This significant difference was also seen at the cut-off of 20% but not at cut-off 15% (*p* = 0.020 and *p* = 0.066, respectively; Supplementary Table S1). Further, significant differences were seen in the HER2 immunohistochemical status and HER2 status in the primary setting in patients with *PIK3CA* mutations in their relapse tumor as compared to those without mutations (*p* = 0.015 and *p* = 0.011, respectively; Supplementary Table S1 and Supplementary Fig. S3a-b). Finally, among relapse tumors, distant metastases and contralateral tumors without DCIS showed a significantly higher frequency of *PIK3CA* mutations (*p* = 0.037; Supplementary Table S1 and Supplementary Fig. S4).

No significant differences were seen when comparing the presence of *PIK3CA* mutations in the primary and relapse tumors to the expression of ER or PR in primary and relapse tumors (Table 1 and Supplementary Table S1). However, when comparing steroid receptor expression between patients primary and relapse tumors, a significant difference was seen in PR (*p* = 0.0049) where relapse tumors tended to have lower expressions, but not for ER (*p* = 0.80).

### *PIK3CA* mutational status

**Figure 1:**
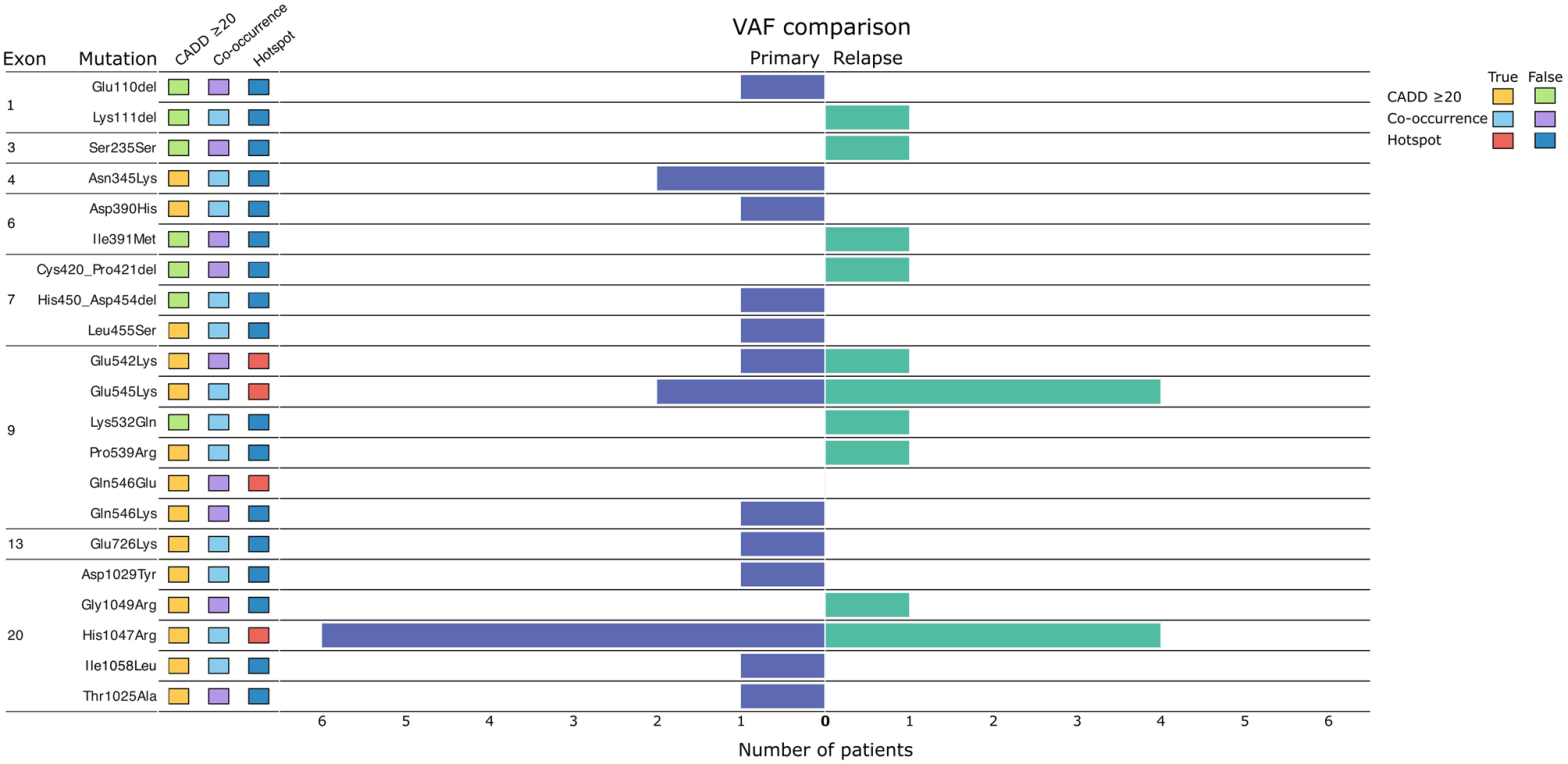
Heatmap and barplot of the *PIK3CA* mutations and subsequent information of the cohorts’ primary and relapse tumors. Exon location, mutation variants, Combined Annotation-Dependent Depletion (CADD) score evaluation (yellow = CADD-score equal to or above 20, green = CADD-score below 20), status of co-occurrence with other mutations (light-blue = co-occurs with other *PIK3CA* mutations, light-purple = does not co-occur with other *PIK3CA* mutations), hotspot status (red = hotspot mutation, blue non-hotspot mutation), and variant allele frequency (VAF) comparisons on X-axis (number of patients in which the VAF is higher in either tumor sample; purple = VAF higher in primary tumor than relapse tumor, green = VAF higher in relapse tumor than primary tumor) for each of the individual mutations found. NA = comparison not possible (left blank), orphan samples.

*PIK3CA* mutations were present in 50% (31/62) of the patients. Out of these, 25 patients had one single mutation, whereas six patients had two or more mutations. Analysis of mutational status was further carried out on the 58 primary and 54 relapse tumors where sequencing data was available. Mutations were present in 40% (23/58) of primary tumors, and 48% (26/54) of relapse tumors. Hotspot mutations were found in 35% (22/62) of all patients and in 71% (22/31) of the patients with mutations. Among the mutated primary tumors, 65% (15/23) had hotspot mutations, accounting for 26% (15/58) of the overall primary tumors. Hotspot mutations were observed in 33% (18/54) of all relapse tumors and in 69% (18/26) of mutated relapse tumors. The *PIK3CA* mutations and information on exon location, hotspot status, CADD-scores, co-occurrence with other mutations, and VAF comparisons are illustrated in Fig. 1 and described further below. The mutations are further summarized with information, including Human Genome Variation Society (HGVS) nomenclature, consequence, ClinVar definition, CADD-score, COSMIC and dbSNP identification, and information on presence in other studies in Supplementary Table S2.

In total, 21 mutations were found in 49 of the total 112 tumor samples (44%), of which 17 were non-hotspot mutations. The most common alterations were the hotspot mutations His1047Arg, accounting for 27% (11/41) of the mutations, followed by Glu545Lys in 22% (9/41). Among the 41 mutations, 35 mutations could be evaluated in both primary and relapse tumors of the same patient, while the remaining six were only present in patients’ orphan primary or relapse samples. Of these mutations, 54% (19/35) were retained between the primary and relapse tumor, whereas 20% (7/35) of the mutations were lost from primary to relapse, and 26% (9/35) were gained in the relapse setting and not seen in the primary tumor sample. Of all the mutations, 37% (15/41) occurred on exon 9 and 20, respectively, and 7% (3/41) on exon 7. The remaining exons included 5% (2/41) of mutations on exons 1, 4, and 6, respectively, and 2% (1/41) on exons 3 and 13. CADD scores showed that 67% (14/21) of the mutations had a score at or above 0.20. Of the non-hotspot mutations, 41% (7/17) had CADD scores < 0.20 or not available (NA).

**Figure 2:**
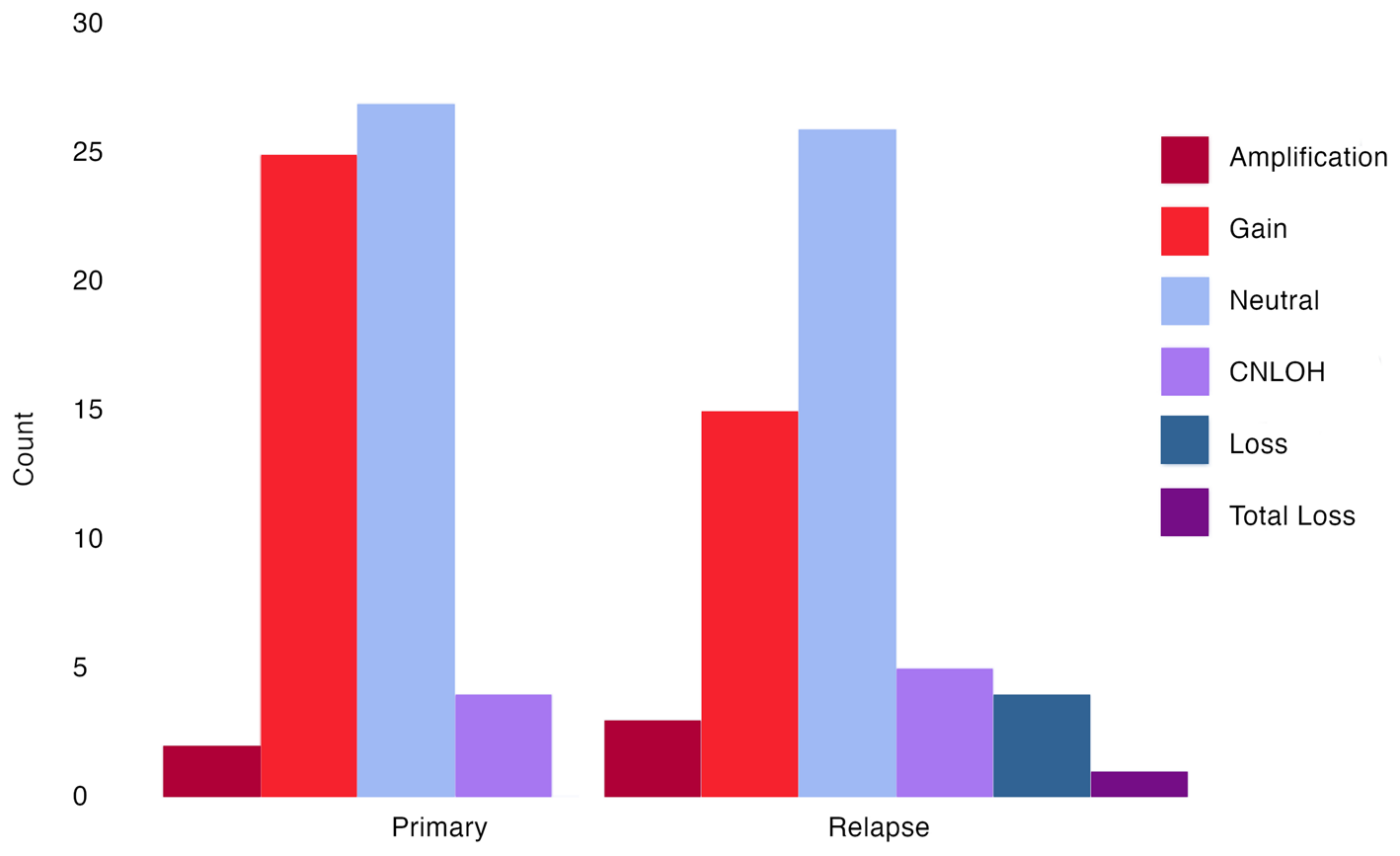
Histogram of the frequencies of *PIK3CA* copy number variations in the cohorts’ primary and relapse tumors. CNLOH = copy-neutral loss of heterozygosity.

FACETS copy number analysis revealed variations for the *PIK3CA* gene in 65% (40/62) of patients; in 53% (31/58) of primary tumors and in 52% (28/54) of relapse tumors. For the patients' primary tumors, 3% (2/58) had amplifications, 43% (25/58) had gains, 7% (4/58) had a copy-neutral loss of heterozygosity (CNLOH), and 47% (27/58) had neutral copy numbers. In the relapse tumors, 6% (3/54) had amplifications, 28% (15/54) had gains, 9% (5/54) CNLOH, 48% (26/54) neutral status, 7% (4/54) a loss and 2% (1/54) a total loss (Fig. 2). Out of the 50 patients with a complete pair of tumors, 19 patients had copy number variations in both the primary and relapse tumors, where 12 retained amplifications and gains in both tumors. Five pairs gained variations in the relapse setting and seven pairs lost their variation to a neutral status.

### Survival analyses and mutational status

**Figure 3:**
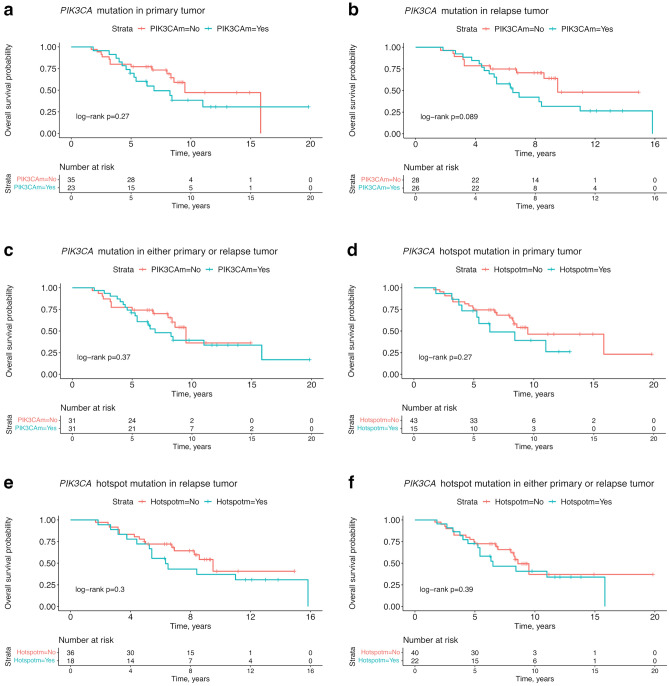
Overall survival analysis of the patients in the cohort compared to their different *PIK3CA* mutational status. Kaplan–Meier estimates for overall survival of patients with tumors harboring a *PIK3CA* mutation in the primary tumor (**a**), relapse tumor (**b**), or in any of the paired tumor samples (**c**). Kaplan–Meier estimates for overall survival of patients with tumors harboring a *PIK3CA* hotspot mutation in primary tumors (**d**), relapse tumors (**e**), or in any of the tumor samples (**f**). *PIK3CA*m = *PIK3CA* mutation, Hotspotm = *PIK3CA* hotspot mutation.

**Figure 4:**
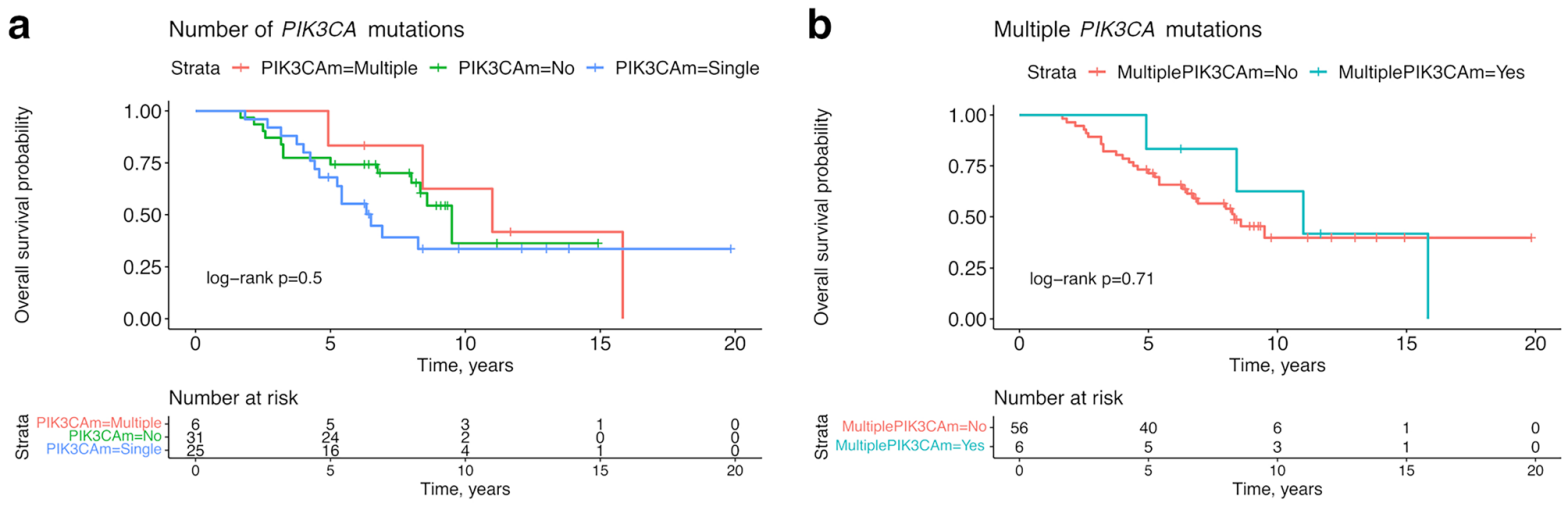
Overall survival analyses of the cohort assessed by the number of *PIK3CA* mutations. Kaplan–Meier estimates for overall survival of patients with tumors harboring a single, multiple, or no *PIK3CA* mutations in any tumor sample (**a**), and of patients with tumors harboring multiple *PIK3CA* mutations or not (single or no mutation) (**b**). *PIK3CA*m = *PIK3CA* mutation.

In survival analyses with Kaplan–Meier estimates, no significant associations were found between groups with different mutational statuses and overall survival (Fig. 3). No significant association with overall survival was seen when assessing *PIK3CA* mutations in the primary (log-rank *p* = 0.27; Fig. 3a) or relapse tumor (log-rank *p* = 0.089; Fig. 3b), or if present in either the primary or relapse tumor (log-rank *p* = 0.37; Fig. 3c). Similarly, there was no significant difference in overall survival between patients with or without *PIK3CA* hotspot mutation in the primary tumor (log-rank *p* = 0.27; Fig. 3d), nor in the relapse tumor (log-rank *p* = 0.3; Fig. 3e), or in any tumor sample (log-rank *p* = 0.39; Fig. 3f). Further, there was no significant difference in overall survival when comparing patients with multiple, single, or no *PIK3CA* mutations (log-rank *p* = 0.5; Fig. 4a) as well as when comparing multiple *PIK3CA* mutations to either a single or no mutation (log-rank *p* = 0.71; Fig. 4b). Similarly, for all analyses of breast cancer-specific survival, none of the above-mentioned comparisons showed significant associations (Supplementary Fig. S5 and Supplementary Fig. S6).

### Associations between *PIK3CA* mutations and lymph node status

**Figure 5:**
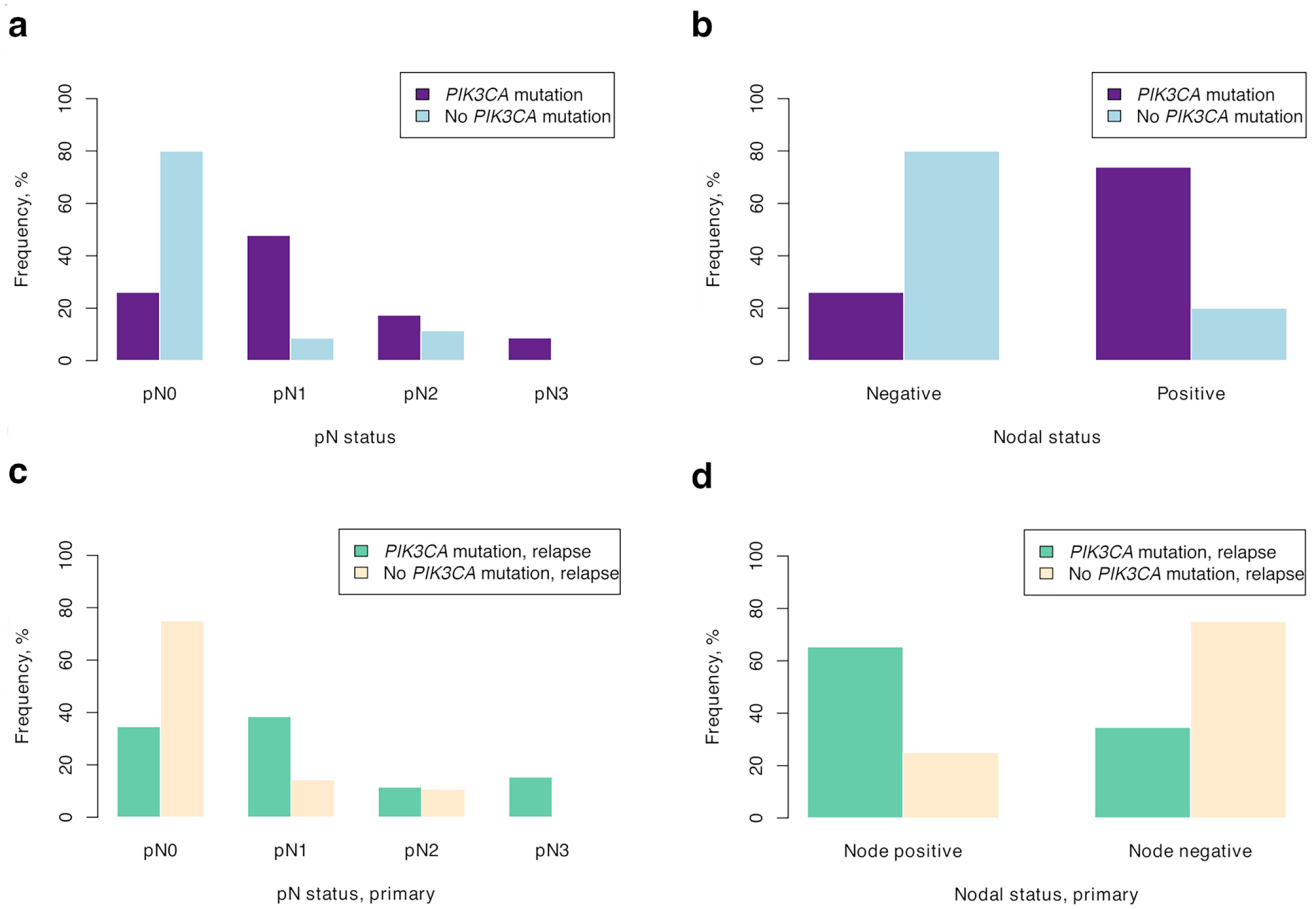
Distribution of *PIK3CA* mutational status in patients’ tumors across nodal status in the primary setting. Distribution of *PIK3CA* mutational status in primary tumors by pN category (**a**) and nodal status (**b**) of the primary tumor. Distribution of mutational status of relapse tumors by pN category (**c**) and nodal status (**d**) of the primary tumor.

The presence of *PIK3CA* mutations in primary tumors was significantly associated with lymph node metastasis in the primary setting, both when assessing nodal status as negative (pN0) versus positive (pN1-3), and the respective pN category (pN0-3; Table 1). Patients with *PIK3CA* mutations in the primary setting had higher pN-status (*p* = 0.000070) and a higher proportion were node-positive (*p* = 0.000088), as compared to patients without a *PIK3CA* mutation (Fig. 5a,b). Further, patients with hotspot mutations in the primary setting showed similar results with pN status and nodal status. In the relapse setting, the associations were also statistically significant when comparing *PIK3CA* mutations in the relapse tumor to the nodal status and pN status in the primary tumor setting (*p* = 0.0056 and *p* = 0.0064, respectively; Fig. 5c,d). This was also shown for the hotspot mutations in the relapse tumor compared to the nodal status in the primary tumor, but not compared to pN status. However, there were no significant associations of nodal status in the relapse setting compared to relapse mutational status (*p* = 1), nor for hotspot status.

## Discussion

PI3K inhibitors have conveyed promising results in the treatment of patients with endocrine-resistant breast cancer. Assessing *PIK3CA* mutations is highly relevant, and an improved outcome has been shown for patients harboring the hotspot mutations and treated with PI3K inhibitors. Yet the prevalence of *PIK3CA* mutations, hotspot or not, in relapse tumors during treatment compared to primary tumors, and how the tests are carried out, remain as questions. Further, different studies have found disparate results when assessing clinical parameters in association with *PIK3CA* mutations, where some find significance for both positive and negative predictive factors. This study has shed light upon the differential *PIK3CA* mutational profiles between relapse and primary tumors, and their associations to clinicopathological variables, especially lymph node status, in a unique paired cohort of patients with confirmed endocrine-resistant breast cancer.

Half (50%) of the patients carried a *PIK3CA* mutation either in the primary or relapse tumor and the mutational frequency was higher in the relapse setting (48%) than in the primary (40%). Non-hotspot mutations were common, and even more so in primary tumors (29% in either tumor, 35% in primary and 31% in relapse tumors of patients with *PIK3CA* mutations). A previous study on metastatic ER + /HER2- breast cancer showed moderately lower frequencies (35%) of patients having a *PIK3CA* mutation when assessing with NGS techniques, and out of these 20% had non-hotspot mutations^22^. In an analysis of 10 datasets of breast tumors, a 20% frequency of non-hotspot mutations has also been reported^21^. This could indicate that relapse tumors during ongoing endocrine treatment more frequently carry mutations, and out of these, almost one-third, seem to harbor non-hotspot mutations, which would go unnoticed by the hotspot testing. Perhaps the treatment may favor mutational activity, and even more so in hotspot regions. At the same time, the lower frequency of mutations outside hotspot regions in the relapse tumors could suggest a possible treatment effect. Previously, a significant change in *PIK3CA* mutational frequency has not been shown for patients with treatment-naïve distant metastases compared to treatment-naïve primary tumors^46^, whereas an increase has been seen in early-course and predominantly treatment-naïve metastases^47^. These findings may support the theory that *PIK3CA* mutations are more prevalent during endocrine treatment, thus early in the disease progression. This highlights that it is crucial that the tumor relapse or metastasis is assessed for mutational status in the advanced setting, otherwise, possible beneficial treatment may not be offered to the patient. Even though the study sample is limited, the results suggest that 54% of the *PIK3CA* mutations were retained between the patients primary and relapse tumor, possibly indicating that a test could be beneficial already at the primary setting. However, the remaining mutations in this cohort were either lost or gained. In addition, it is not verified if additional testing and treatment at the primary setting would benefit the patients but could be of interest to investigate further.

Evaluating the functionality and clinical consequence of mutations is challenging^48,49^. Several resources and databases were included in the analysis to assess the potential clinical effects of the mutations outside hotspot regions. The Consensus Coding Sequences (CCDS) annotation was used^50^ and evaluations involved COSMIC and dbSNP comparisons, previous studies in the field, and CADD-score assessments. Noteworthy, only four (His1047Arg, Glu545Lys, Glu542Lys, and Gln546Glu) of the 11 hotspot mutations that are indicated in the PIQRAY CDx QIAGEN Therascreen® PIK3CA test were found in our cohort, but thus various others. In a study of combined hotspot analyses of primary untreated tumors in The Cancer Genome Atlas (TCGA) program with endocrine-resistant tumors, *PIK3CA* mutations were shown as significantly mutated, and 15 novel hotspot mutations were suggested. One of these was the Glu110del mutation, also present in our cohort, which was seen to induce the PI3K pathway in cell lines^46^. Other mutated *PIK3CA* variations in the study similar to ours involved Asn345Lys, Cys420_Pro421del, Gln546Lys, Pro539Arg, Glu726Lys, Gly1049Arg, and Thr1025Ala^46^, suggesting that these alterations indeed have clinical relevance in the endocrine-resistant setting and should be considered for treatment assessment. Further, a study that compared the *PIK3CA* mutations in all publicly available datasets of breast cancers, including 6477 samples from 10 studies, showed the mutations Glu110del, Asn345Lys, Gln546Lys, Glu726Lys, and Gly1049Arg, which are also present in our cohort^21^. Research comparing comprehensive genomic profiling to the Therascreen® mutations demonstrated that the majority of patients' tumors harbored the hotspot mutations^22^. Similarly to our results, mutations such as Glu110del, Asn345Lys, Gln546Lys, Pro539Arg, Glu726Lys, and Gly1049Arg were present in the advanced setting^22^. Several of these mutations have been found to be of relevance in pre-clinical studies^21,51^. The comparative study of Therascreen® mutations also presented a longer median real-world progression-free survival for patients with mutations outside of the hotspot regions that received alpelisib combined with fulvestrant as compared to those receiving fulvestrant alone^22^.

Multiple mutations were present in six patients in our study, supporting the idea of a hypermorphic phenotype with one more oncogenic variant followed by a less oncogenic one ^24^. Yet the survival analyses did not show a significant association with overall survival when comparing the number of *PIK3CA* mutations (*p* = 0.5), although the number of patients was low in our study. Further, copy number analysis revealed that copy number alterations were present in 40 patients, whereas mutations were evident in 31 patients. Similarly, 31 primary tumors and 28 relapse tumors had CNVs as compared to the mutations in 23 and 26 of these, respectively. CNVs in *PIK3CA* thus seem to be even more common than the SNVs, especially in primary tumors, consistent with previous research^52^. Unfortunately, allele-specific mutation assessment of the six patients was not technically possible due to the locations of heterozygous single nucleotide polymorphisms being too far apart to share reads with the SNVs.

The most evident clinicopathological difference between the patients with and without a *PIK3CA* mutation was nodal status. A relationship could be seen in the relapse and primary tumors, both on hotspot mutations alone and with all *PIK3CA* mutations, where having a mutation was associated with a higher pN status and node positivity in the primary setting. This suggests that the *PIK3CA* mutated tumors may be more aggressive and spread to lymph nodes. However, some patients without nodal involvement were also seen with *PIK3CA* mutations and relapse during ongoing treatment. A previous study on 292 primary breast cancers demonstrated associations of lymph node spread and hormonal receptor positivity with *PIK3CA* mutations^14^, albeit, other studies have not confirmed this association^15,53^, and in Tunisian patients, a negative association was shown^54^. In these studies, it is not possible to deduct eventual endocrine-resistance development, and our results could thus suggest that nodal involvement together with the presence of a *PIK3CA* mutation is of greater importance in patients who develop endocrine resistance. A theory could thus be that the patients with ER + /HER2- breast cancers and nodal involvement already in the primary setting may have a greater risk of harboring a *PIK3CA* mutation and developing resistance to treatment, and could thus be prioritized for mutational testing.

To our knowledge, no other study describes the relationship of *PIK3CA* mutations in patients with verified endocrine-resistant relapses during ongoing therapy compared to their primary tumors. This is of interest to investigate since treatments are being introduced into the clinical setting together with accompanying tests, and there is a demand for comprehension of the mutational statuses to support understanding of the resistance mechanism(s). This study, however, involved stringent inclusion criteria to retrieve a specific cohort with intricate characteristics which, in turn, led to a smaller cohort than the actual probable number of endocrine-resistant patients in Stockholm during the studied years. Nonetheless, the specificity of the cohort also decreases possible confounding factors and may aid in the interpretation of the results. Extensions of the study are needed in order to draw general conclusions and advance the treatment indications for a greater number of patients with breast cancer. Since treatment compliance is a major issue for patient outcomes and therapy evaluation^55^, we assessed treatment compliance in patients’ medical records. Further, it is generally known that using FFPE tissue for extraction and sequencing is a delicate procedure and may impact the quality of the genetic material. The panel sequencing used has proven to be a robust method for this type of material, and thus from the samples that passed preparation, it is possible to analyze comprehensible results^25,26^. This study focused solely on the *PIK3CA* mutations in a verified endocrine-resistant cohort. However, several other mutations have shown importance and involvement in the resistance development, where for example investigations of *ESR1* and *TP53* could aid in assessments of intrinsic and acquired resistance ^8,46,47^. Further, alterations in receptor tyrosine kinases such as members of the epidermal growth factor and fibroblast growth factor receptor families are suggested mechanisms of endocrine resistance and may be relevant to examine in this cohort^4,8^. Moreover, loss of function mutations in *PTEN* (phosphatase and tensin homolog), also demonstrated to be mutually exclusive with *PIK3CA* mutations in BC, and mutations in *AKT1* (AKT serine/threonine kinase 1) and *NF1* (neurofibromin 1) are associated with endocrine resistance and could be valuable extended assessments of these results^4,8,14^. Analysis of the entire sequenced panel is performed in an ongoing study, to further explore differences between intrinsic and acquired resistance and to visualize the broader mutational landscape of the cohort. Moreover, mutual exclusivity and synergistic mutations may be evaluated together with associations of the different involved pathways, which can give further biological insight into the resistance mechanism.

In conclusion, this study indicates that differential *PIK3CA* mutations are evident in patients with verified endocrine-resistant breast cancer tumors and are associated with lymph node involvement. The mutations were more frequent in the relapse tumor setting during ongoing treatment, further supporting that testing of *PIK3CA* mutation status for PI3K inhibitor treatment should be carried out on the advanced tumor specimen for treatment in the advanced setting. The abundance of mutations occurring outside of hotspot regions implies that there also is a need for expanding the assessment and not solely testing for the hotspots. Our results may help further investigations of *PIK3CA* mutational status in patients with endocrine-resistant breast cancer, which can aid in optimizing their treatment and subsequent assessment. However, the specific findings need to be investigated further and specifically analyzed in a larger cohort.

### Supplementary Information

Supplementary Information.

## Data Availability

The raw sequencing data sets analyzed in the current study are uploaded to The European Genome-phenome Archive (EGA) under accession number EGAS50000000236 (https://ega-archive.org/studies/EGAS50000000236).
