# Supplementary material for: PIK3CA mutations in endocrine-resistant breast cancer
Source: Sci Rep. 2024 May 31;14:12542. doi: 10.1038/s41598-024-62664-1 (PMC11143214; doi:10.1038/s41598-024-62664-1)
Supplement: Supplementary file 1 — Supplementary Information. [file 41598_2024_62664_MOESM1_ESM.pdf]

Supplementary Information

PIK3CA mutations in endocrine-resistant breast cancer

Caroline Schagerholm, Stephanie Robertson, Hosein Toosi, Emmanouil G. Sifakis, Johan Hartman

Corresponding author: Caroline Schagerholm, MD. Department of Oncology and Pathology, Karolinska Institutet, Sweden. E-mail: caroline.schagerholm@ki.se

Table of contents

Supplementary Tables:

Table S1. Patient and tumor characteristics of the cohort, divided on *PIK3CA* mutations in the relapse tumor.
Table S2. Overview of the *PIK3CA* mutations in the cohort, including HGVS nomenclature, clinical measures, database information, and presence in other studies for non-hotspot mutations.

Supplementary Figures:

Figure S1. Consort diagram describing the selection of patients in the Endoresist cohort.
Figure S2. Boxplots and barplots of *PIK3CA* mutational status in primary and relapse tumors compared to Ki67 status.
Figure S3. Barplots of *PIK3CA* mutational status of relapse tumors compared to HER2 status of the primary tumors.
Figure S4. Barplot of the distribution of *PIK3CA* mutational status in relapse tumors compared to the relapse location.
Figure S5. Breast cancer specific survival analysis of the patients in the cohort compared to their different *PIK3CA* mutational status.
Figure S6. Breast cancer specific survival analyses of the cohort assessed by the number of *PIK3CA* mutations.

Table S1. Patient and tumor characteristics of the cohort, divided on *PIK3CA* mutations in the relapse tumor.

	All	Relapse tumor with *PIK3CA*	Relapse tumor with no *PIK3CA* mutation	P-value mutation (mutation vs no mutation per characteristic)
	**N=62***	**N=26****	**N=28****	
**Age at primary diagnosis**				P=0.40
Median, years (range)	61 (30-88)	62 (31-80)	60.5 (30-88)	
**Histological subtype primary**				P=0.26
Ductal	43 (69.35%)	15 (57.69%)	22 (78.57%)	
Lobular	14 (22.58%)	8 (30.77%)	5 (17.86%)	
Other	5 (8.06%)	3 (11.54%)	1 (3.57%)	
**Histological subtype relapse**				P=0.62
Ductal	30 (48.39%)	10 (38.46%)	17 (60.71%)	
Lobular	11 (17.74%)	5 (19.23%)	4 (14.29%)	
Other	2 (3.23%)	1 (3.85%)	1 (3.57%)	
NA	19 (30.65%)	10 (38.46%)	6 (21.43%)	
**Tumor size primary**				P=0.70
Median, mm (range)	24 (10-100)	23 (10-60)	25 (10-100)	
**Tumor size relapse**				P=0.14
Median, mm (range)	14 (2-75)	19.5 (4-75)	12.5 (2-55)	
**Tumor stage primary**				P=0.51
pT1	23 (37.10%)	9 (34.62%)	9 (32.14%)	
pT2	34 (54.84%)	16 (61.54%)	15 (53.57%)	
pT3	5 (8.06%)	1 (3.85%)	4 (14.29%)	
**Histologic grade primary**				P=0.93
NHG1	9 (14.52%)	4 (15.38%)	3 (10.71%)	
NHG2	28 (45.16%)	12 (46.15%)	13 (46.43%)	
NHG3	24 (38.71%)	10 (38.46%)	12 (42.86%)	
NA	1 (1.61%)	0 (0.00%)	0 (0.00%)	
**Histologic grade relapse**				P=0.069
NHG1	5 (8.06%)	2 (7.69%)	2 (7.14%)	
NHG2	17 (27.42%)	8 (30.77%)	6 (21.43%)	
NHG3	14 (22.58%)	2 (7.69%)	11 (39.29%)	
NA	26 (41.94%)	14 (53.85%)	9 (32.14%)	
**pN stage primary**				P=0.0064
pN0	35 (56.45%)	9 (34.62%)	21 (75.00%)	
pN1	15 (24.19%)	10 (38.46%)	4 (14.29%)	
pN2	8 (12.90%)	3 (11.54%)	3 (10.71%)	
pN3	4 (6.45%)	4 (15.38%)	0 (0.00%)	
**Lymph node status primary**				P=0.0056
Node negative	35 (56.45%)	9 (34.62%)	21 (75.00%)	
Node positive	27 (43.55%)	17 (65.38%)	7 (25.00%)	
**Lymph node status relapse**				P=1
Node negative	20 (32.26%)	6 (23.08%)	11 (39.29%)	
Node positive	11 (17.74%)	4 (15.38%)	7 (25.00%)	
NA	31 (50.00%)	16 (61.54%)	10 (35.71%)	
**TNM stage primary**				P=0.26
Stage 1	16 (25.81%)	5 (19.23%)	9 (32.14%)	
Stage 2	34 (54.84%)	14 (53.85%)	16 (57.14%)	
Stage 3	12 (19.35%)	7 (26.92%)	3 (10.71%)	
**ER, % primary**				P=0.14
Median, % (range)	90 (5-100)	90 (50-100)	90 (5-100)	
**ER, % relapse**				P=0.14
Median, % (range)	90 (20-100)	90 (20-100)	95 (50-100)	
**PR, % primary**				P=0.43
Median, % (range)	40 (0-100)	32.5 (0-100)	60 (0-100)	
**PR status primary**				P=0.51
<10%	14 (22.58%)	7 (26.92%)	5 (17.86%)	
≥10%	35 (56.45%)	14 (53.85%)	17 (60.71%)	
NA	13 (20.97%)	5 (19.23%)	6 (21.43%)	
**PR status primary**				P=0.33
<20%	16 (25.81%)	8 (30.77%)	5 (17.86%)	
≥20%	33 (53.23%)	13 (50.00%)	17 (60.71%)	
NA	13 (20.97%)	5 (19.23%)	6 (21.43%)	
**PR, % relapse**				P=0.77
Median, % (range)	5 (0-100)	5 (0-100)	5 (0-100)	
**PR status relapse**				P=1
<10%	27 (43.55%)	11 (42.31%)	14 (50.00%)	
≥10%	23 (37.10%)	9 (34.62%)	11 (39.29%)	
NA	12 (19.35%)	6 (23.08%)	3 (10.71%)	
**PR status relapse**				P=0.76
<20%	33 (53.23%)	12 (46.15%)	17 (60.71%)	
≥20%	17 (27.42%)	8 (30.77%)	8 (28.57%)	
NA	12 (19.35%)	6 (23.08%)	3 (10.71%)	

**Ki67, % primary**				P=0.028
Median, % (range)	20 (1-95)	14.5 (1-80)	20 (5-95)	
**Ki67 status primary**				P=0.066
<15%	20 (32.26%)	11 (42.31%)	6 (21.43%)	
≥15%	34 (54.84%)	10 (38.46%)	20 (71.43%)	
NA	8 (12.90%)	5 (19.23%)	2 (7.14%)	
**Ki67 status primary**				P=0.020
<20%	25 (40.32%)	14 (53.85%)	8 (28.57%)	
≥20%	29 (46.77%)	7 (26.92%)	18 (64.29%)	
NA	8 (12.90%)	5 (19.23%)	2 (7.14%)	
**Ki67, % relapse**				P=0.89
Median, % (range)	20 (0-100)	25 (1-60)	20 (0-100)	
**Ki67 status relapse**				P=0.74
<15%	13 (20.97%)	6 (23.08%)	6 (21.43%)	
≥15%	40 (64.52%)	15 (57.69%)	21 (75.00%)	
NA	9 (14.52%)	5 (19.23%)	1 (3.57%)	
**Ki67 status relapse**				P=0.56
<20%	18 (29.03%)	8 (30.77%)	8 (28.57%)	
≥20%	36 (58.06%)	13 (50.00%)	19 (67.86%)	
NA	8 (12.90%)	5 (19.23%)	1 (3.57%)	
**HER2 IHC primary**				P=0.015
0	37 (59.68%)	20 (76.92%)	12 (42.86%)	
1+	11 (17.74%)	4 (15.38%)	5 (17.86%)	
2+	9 (14.52%)	1 (3.85%)	8 (28.57%)	
3+	2 (3.23%)	0 (0.00%)	2 (7.14%)	
NA	3 (4.84%)	1 (3.85%)	1 (3.57%)	
**HER2 IHC relapse**				P=0.39
0	28 (45.16%)	10 (38.46%)	16 (57.14%)	
1+	19 (30.65%)	9 (34.62%)	7 (25.00%)	
2+	9 (14.52%)	5 (19.23%)	3 (10.71%)	
3+	1 (1.61%)	0 (0.00%)	1 (3.57%)	
NA	5 (8.06%)	2 (7.69%)	1 (3.57%)	
**HER2 status primary*****				P=0.011
HER2-negative/zero	37 (59.68%)	20 (76.92%)	12 (42.86%)	
HER2-low	22 (35.48%)	5 (19.23%)	15 (53.57%)	
HER2-positive	0 (0.00%)	0 (0.00%)	0 (0.00%)	
NA	3 (4.84%)	1 (3.85%)	1 (3.57%)	
**HER2 status relapse*****				P=0.21
HER2-negative/zero	28 (45.16%)	10 (38.46%)	16 (57.14%)	
HER2-low	28 (45.16%)	14 (53.85%)	10 (35.71%)	
HER2-positive	1 (1.61%)	0 (0.00%)	1 (3.57%)	
NA	5 (8.06%)	2 (7.69%)	1 (3.57%)	
**TIL-scoring primary**				P=0.86
Median, % (range)	5 (1-40)	5 (1-40)	5 (1-35)	
**TIL-scoring relapse**				P=0.61
Median, % (range)	10 (1-40)	10 (1-30)	5 (1-40)	
**Surgical procedure primary**				P=0.78
Mastectomy	30 (48.39%)	14 (53.85%)	14 (50.00%)	
Partial mastectomy	31 (50.00%)	11 (42.31%)	14 (50.00%)	
NA	1 (1.61%)	1 (3.85%)	0 (0.00%)	
**Adjuvant endocrine therapy**				P=0.25
Tamoxifen	33 (53.23%)	10 (38.46%)	17 (60.71%)	
Aromatase inhibitor	24 (38.71%)	13 (50.00%)	9 (32.14%)	
Other	5 (8.06%)	3 (11.54%)	2 (7.14%)	
**Adjuvant chemotherapy**				P=0.061
Received	32 (51.61%)	18 (69.23%)	12 (42.86%)	
Not received	30 (48.39%)	8 (30.77%)	16 (57.14%)	
**Adjuvant radiotherapy**				P=0.17
Received	40 (64.52%)	19 (73.08%)	15 (53.57%)	
Not received	22 (35.48%)	7 (26.92%)	13 (46.43%)	
**Relapse location**				P=0.037
Ipsilateral	22 (35.48%)	7 (26.92%)	11 (39.29%)	
Contralateral without DCIS	5 (8.06%)	4 (15.38%)	1 (3.57%)	
Contralateral with DCIS	14 (22.58%)	3 (11.54%)	10 (35.71%)	
Distant metastasis	21 (33.87%)	12 (46.15%)	6 (21.43%)	

DCIS=ductal carcinoma in situ, ER=estrogen receptor, IHC=immunohistochemistry, NHG=Nottingham Histologic Grade, pN=pathological nodal status, PR=progesterone receptor, TIL=tumor-infiltrating lymphocyte, TNM=tumor, nodal, metastasis staging, NA=data not available.

* All patients included, even when lacking sequencing data from the relapse tumor.

** Representing the relapse tumors where sequencing data could be generated and evaluated.

*** HER2-negative/zero=HER2 IHC score 0, HER2-low=HER2 IHC score 1-3+ and negative HER2 *in situ* hybridization (ISH), HER2-positive=HER2 amplified by ISH.

Table S2. Overview of the *PIK3CA* mutations in the cohort, including HGVS nomenclature, clinical measures, database information, and presence in other studies for non-hotspot mutations.

HGVSp	HGVSc (ENST00000263967)	Consequence	ClinVar	CADD	COSMIC/dbSNP (non-hotspot)	Present in studies (non-hotspot)
**Glu110del**	3:c.328_330del	Inframe_deletion	NA	NA	NA	(Martínez-Sáez et al, Razavi et al, Rugo et al)
**Lys111del**	3:c.332_334del	Inframe_deletion	NA	NA	NA	
**Ser235Ser**	3:c.705C>T	Synonymous_variant	NA	11.31	COSV55976362, COSV99843414	
**Asn345Lys**	3:c.1035T>A	Missense_variant	pathogenic, likely_pathogenic	24.4	rs121913284, COSV55873276	(Martínez-Sáez et al, Razavi et al, Rugo et al)
**Asp390His**	3:c.1168G>C	Missense_variant	NA	25.5	NA	
**Ile391Met**	3:c.1173A>G	Missense_variant	benign	14.15	rs2230461, COSV55885079	
**Cys420_Pro421del**	3:c.1259_1264del	inframe_deletion	NA	NA	NA	(Razavi et al)
**His450_Asp454del**	3:c.1349_1363del	Inframe_deletion	NA	NA	COSV55900724	
**Leu455Ser**	3:c.1364T>C	Missense_variant	NA	24.4	NA	
**Gln546Glu**	3:c.1636C>G	Missense_variant	pathogenic, likely_pathogenic	22.4	Hotspot mutation	
**Gln546Lys**	3:c.1636C>A	Missense_variant	pathogenic, likely_pathogenic	25.9	rs121913286, COSV55873527	(Martínez-Sáez et al, Razavi et al, Rugo et al)
**Glu542Lys**	3:c.1624G>A	Missense_variant	pathogenic	31	Hotspot mutation	
**Glu545Lys**	3:c.1633G>A	Missense_variant	pathogenic, likely_pathogenic	30	Hotspot mutation	
**Lys532Gln**	3:c.1594A>C	Missense_variant	NA	17.51	rs1387024511	
**Pro539Arg**	3:c.1616C>G	Missense_variant	likely_pathogenic	27.0	rs121913285, COSV55876380	(Razavi et al, Rugo et al)
**Glu726Lys**	3:c.2176G>A	Missense_variant	pathogenic, likely_pathogenic	23.9	rs867262025, COSV55875460	(Martínez-Sáez et al, Razavi et al, Rugo et al)
**Asp1029Tyr**	3:c.3085G>T	Missense_variant	NA	25.1	COSV55889750	
**Gly1049Arg**	3:c.3145G>C	Missense_variant	likely_pathogenic	23.5	rs121913277, COSV55874453	(Martínez-Sáez et al, Razavi et al, Rugo et al)
**His1047Arg**	3:c.3140A>G	Missense_variant	pathogenic	22.5	Hotspot mutation	
**Ile1058Leu**	3:c.3172A>C	Missense_variant	uncertain_ significance	22.3	rs1576950003, COSV55899901	
**Thr1025Ala**	3:c.3073A>G	Missense_variant	pathogenic	23.6	rs397517202, COSV55873252	(Razavi et al)

CADD=Combined Annotation-Dependent Depletion, COSMIC=Catalogue of Somatic Mutations In Cancer, dbSNP=Single Nucleotide Polymorphism Database, HGVSp=Human Genome Variant protein sequence name, HGVSc=Human Genome Variant coding sequence name, NA=data not available.

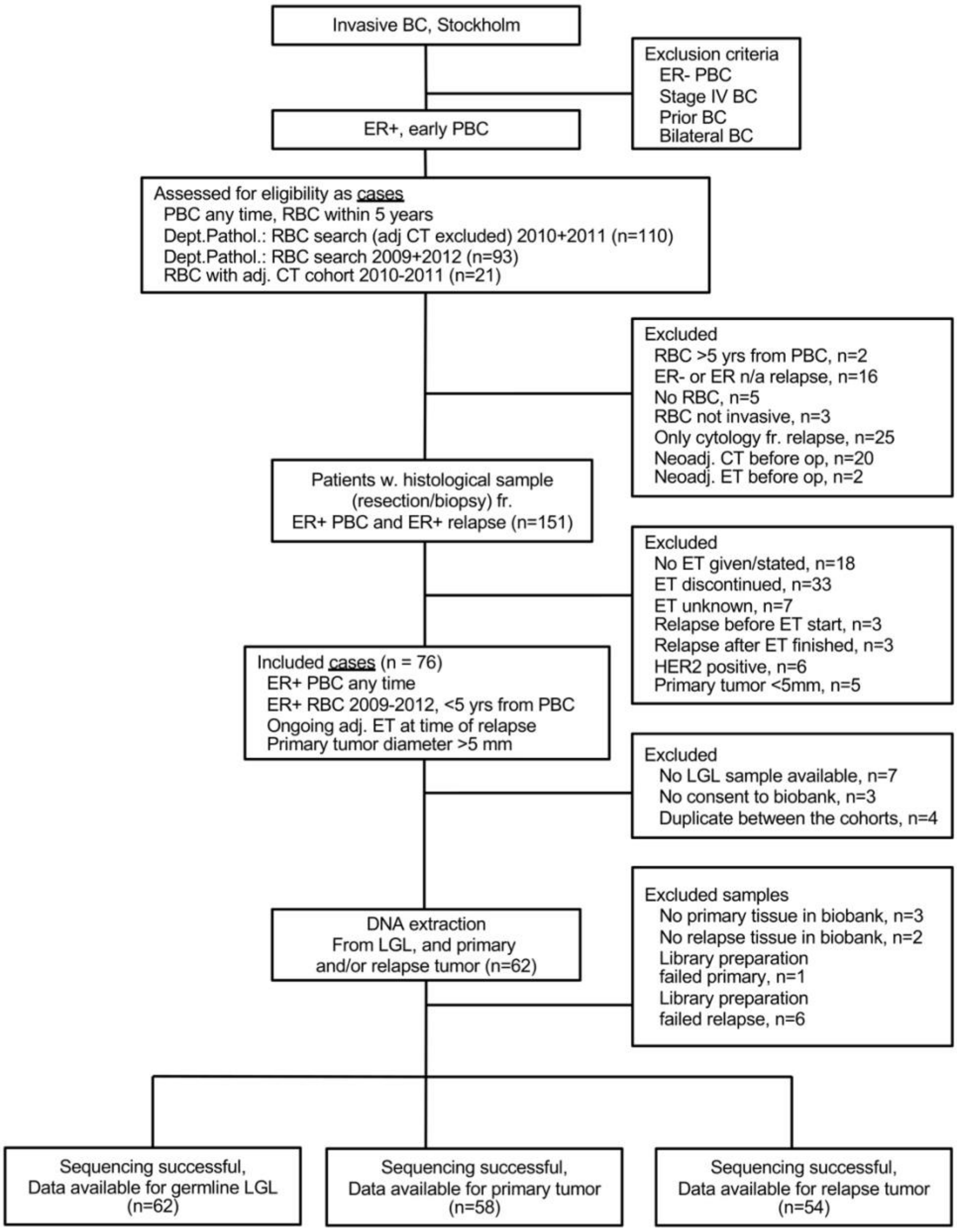

Figure S1. Consort diagram describing the selection of patients in the Endoresist cohort.
Abbreviations: BC=breast cancer, PBC=primary breast cancer, ER+=estrogen receptor-positive, RBC=recurrent breast cancer, CT=chemotherapy, ET=endocrine therapy, F/U = follow-up, LGL=lymph node.

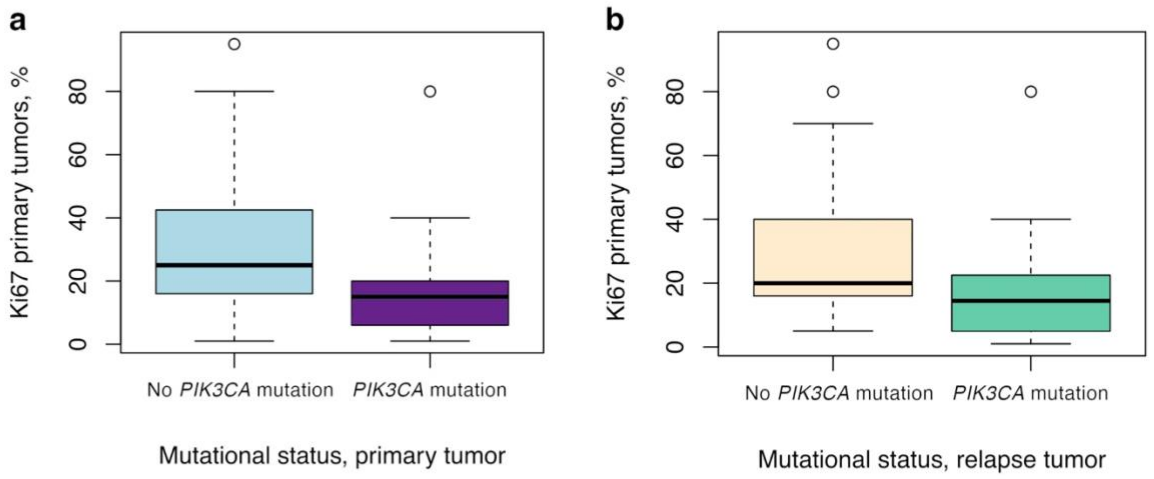

Figure S2. Boxplots and barplots of *PIK3CA* mutational status in primary and relapse tumors compared to Ki67 status. Distribution of relapse *PIK3CA* mutational status compared to Ki67 continuous value in primary (a) and relapse (b) tumors.

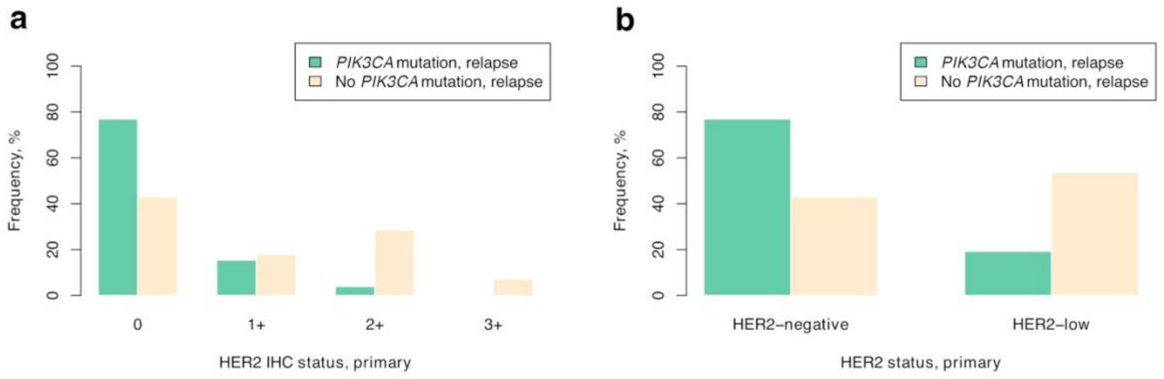

Figure S3. Barplots of *PIK3CA* mutational status of relapse tumors compared to HER2 status of the primary tumors. Distribution of *PIK3CA* mutational status in the relapse tumor across HER2 immunohistochemistry (IHC) score (a) and across HER2 status (b) in the primary tumor.

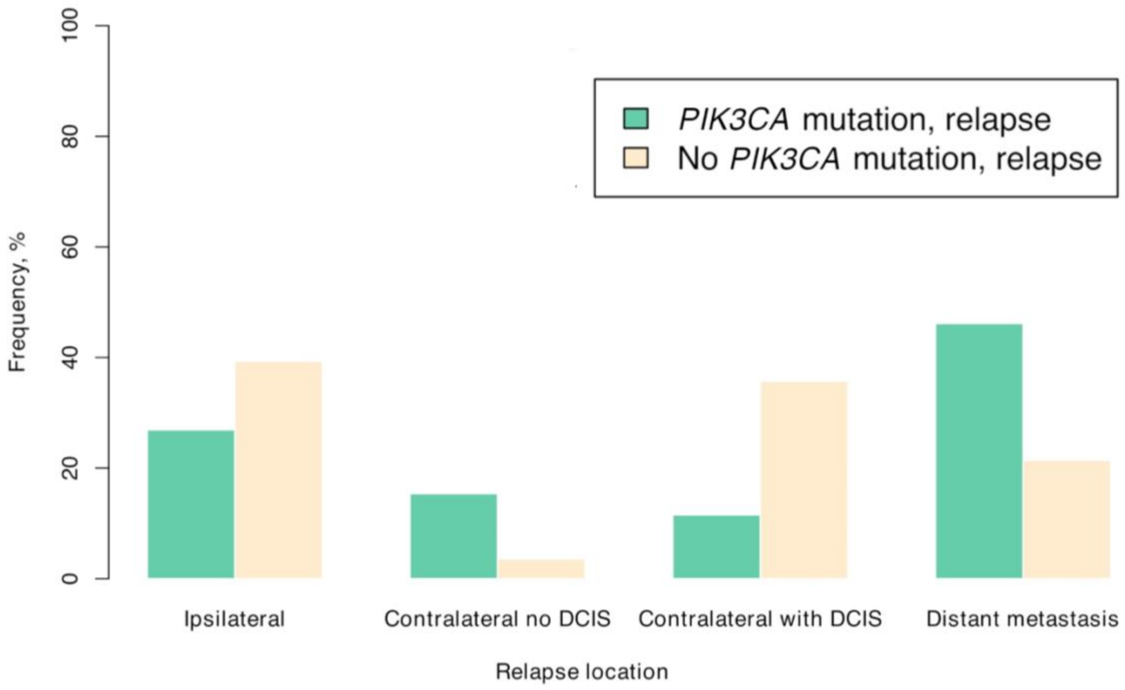

Figure S4. Barplot of the distribution of *PIK3CA* mutational status in relapse tumors compared to the relapse location. DCIS=ductal carcinoma in situ.

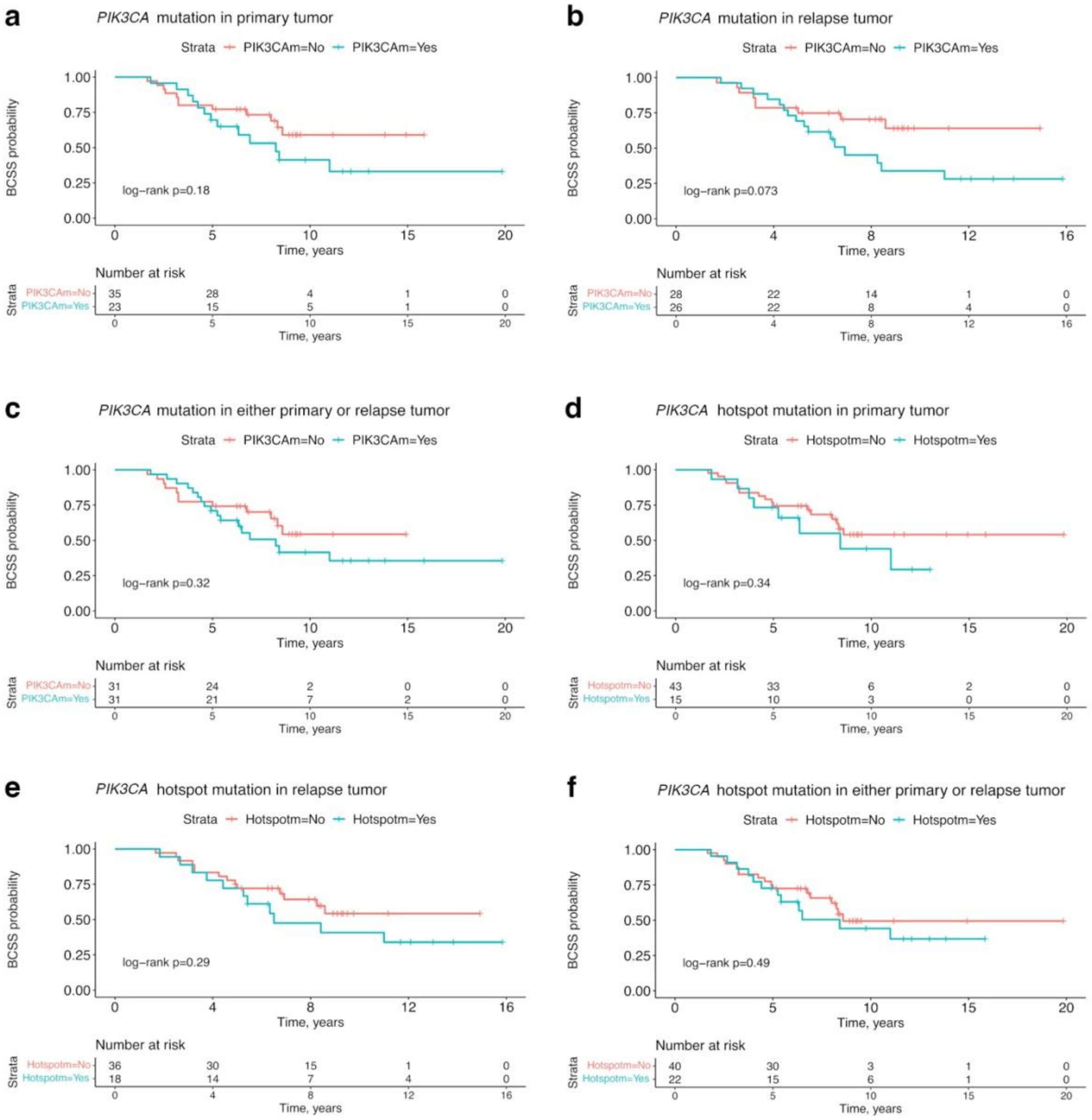

Figure S5. Breast cancer specific survival analysis of the patients in the cohort compared to their different *PIK3CA* mutational status. Kaplan-Meier estimates for breast cancer specific survival (BCSS) of patients with tumors harboring a *PIK3CA* mutation in the primary tumor (a), relapse tumor (b), or in any of the paired tumor samples (c). Kaplan-Meier estimates for BCSS of patients with tumors harboring a *PIK3CA* hotspot mutation in primary tumors (d), relapse tumors (e), or in any of the tumor samples (f). BCSS=breast cancer specific survival, PIK3CAm=*PIK3CA* mutation, Hotspotm=*PIK3CA* hotspot mutation.

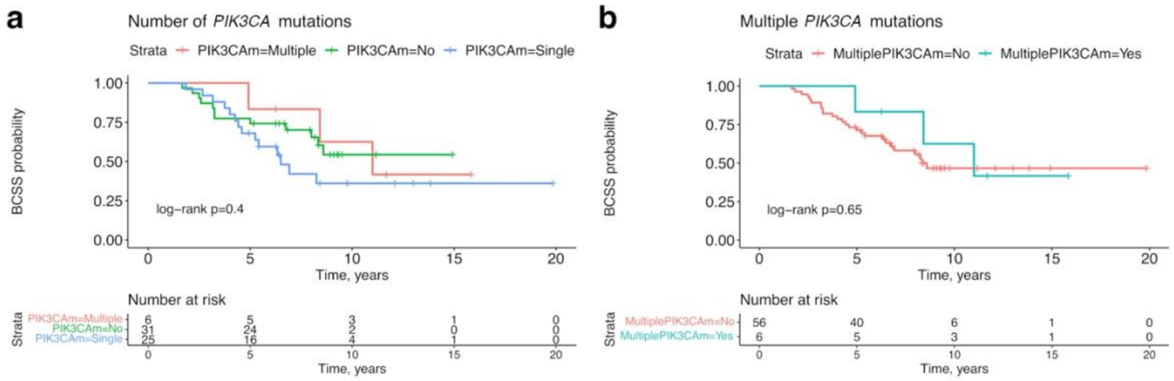

Figure S6. Breast cancer specific survival analyses of the cohort assessed by the number of *PIK3CA* mutations. Kaplan-Meier estimates for breast cancer specific survival (BCSS) of patients with tumors harboring a single, multiple, or no *PIK3CA* mutations in any tumor sample (a), and of patients with tumors harboring multiple *PIK3CA* mutations or not (single or no mutation) (b). BCSS=breast cancer specific survival, PIK3CAm=*PIK3CA* mutation.
